# Meta‐Analysis of Recurrence‐Free Survival or Disease‐Free Survival as a Potential Surrogate Endpoint for Overall Survival in Esophageal Cancer Trials

**DOI:** 10.1002/cnr2.70195

**Published:** 2025-05-19

**Authors:** Uchechukwu Love Anyaduba, Oluwatosin Qawiyy Orababa, Zion Faye, Nazia Rashid, Jason Shafrin, Gregory Reardon

**Affiliations:** ^1^ Henry E. Riggs School of Applied Life Sciences Keck Graduate Institute Claremont California USA; ^2^ School of Life Sciences, Gibbet Hill Campus University of Warwick Coventry UK; ^3^ School of Pharmacy Keck Graduate Institute Claremont California USA; ^4^ Center for Healthcare Economics and Policy FTI Consulting Los Angeles California USA

**Keywords:** disease‐free survival, esophageal cancer, esophageal neoplasms, local, neoplasm recurrence, randomized controlled trial, recurrence, surrogate endpoint

## Abstract

**Background:**

Cancer trials increasingly use surrogate endpoints, but it is unclear how well recurrence‐free survival (RFS) or disease‐free survival (DFS) specifically predict overall survival (OS) in resectable esophageal cancer (EC).

**Methods:**

A systematic literature review identified trials with RFS/DFS and OS endpoints. A meta‐analysis assessed RFS/DFS as surrogates for OS, estimating pooled hazard ratios (HRs) from trial HRs. Forest plots and heterogeneity tests showed effect sizes and pooled estimates. Unweighted linear regression and weighted sensitivity analysis estimated the correlation between OS and RFS/DFS, producing a regression plot.

**Results:**

Of 975 articles identified, 11 met the criteria. The pooled HR for OS and RFS/DFS was 0.90 and 0.87, respectively. The primary analysis showed a strong Pearson correlation between RFS/DFS and OS (*ρ* = 0.89, *p* < 0.001).

**Conclusion:**

Subject to known methodological limits, RFS/DFS was demonstrated to be a potentially suitable surrogate endpoint for OS in resectable EC.

## 1 Introduction

Although the age‐standardized incidence of esophageal cancer (EC) has declined globally over the past 30 years, the rate is increasing in countries that are experiencing rises in life expectancy, education, and income [1, 2]. Cases of the squamous cell carcinoma type of EC are also rising with the aging of the world population [3]. Even with recent advances in treatments, patients with EC currently have only a 20% expected survival rate at 5 years, depending on the stage at diagnosis [4]. To address this poor prognosis, more than 100 EC treatment clinical trials are in progress, positioned at various phases in the pipeline [5].

For late phase randomized controlled trials (RCTs) that evaluate cancer treatments, overall survival (OS), the time from randomization to death arising from any cause, remains the *gold standard* primary endpoint. However, to observe a sufficient number of death events, the use of OS as the primary endpoint requires lengthy subject follow‐up in trials, often 5 years or longer. The U.S. Food and Drug Administration has accepted non‐OS endpoints (e.g., disease‐free survival [DFS], complete response rates) in certain cancer trials as surrogates, reasonably likely to predict clinical benefits like improvements in OS [6]. A given surrogate endpoint for OS could fast‐track treatment clinical testing and regulatory approval, but only if the surrogate is expected to accurately predict the effect of the study treatment on OS.

For EC, clinical endpoints such as progression‐free survival (PFS), recurrence‐free survival (RFS, i.e., first recurrence of cancer) or DFS (i.e., first of recurrence of cancer or death) have been regularly employed in RCTs. Such endpoints might have the potential to serve as candidate surrogates until sufficient confirmatory OS data become available. This study addresses a significant gap in the evaluation of RFS and DFS as surrogate endpoints for OS in resectable EC. Kataoka et al. [7] conducted a meta‐analysis of 10 randomized controlled trials (RCTs) in resectable EC and found weak, nonsignificant correlations between PFS and OS, questioning PFS's validity as a surrogate endpoint. In contrast, Ajani et al. [8] found, in a 2022 meta‐analysis of 26 trials having a mix of either DFS or PFS endpoints in resectable esophageal or gastroesophageal cancer, that DFS/PFS was strongly correlated (i.e., had high predictive ability) with OS. However, both studies have notable limitations. Kataoka et al. focused solely on PFS, while Ajani et al. combined DFS and PFS endpoints without isolating DFS or RFS for independent analysis, which may obscure their specific predictive value.

Within chemotherapy trials for certain cancers like gastric, DFS could serve as a reasonable surrogate endpoint, particularly when chemotherapy is restricted to cytotoxic agents, such as in the adjuvant setting [9]. However, for resectable EC, there has been a lack of meta‐analyses specifically limited to DFS, or recurrence specifically, as surrogate endpoints. Several published clinical trials have defined DFS as encompassing death from any cause, local or regional disease persistence or recurrence, distant metastases, second primary malignancy, or censoring, with the event occurring first [10, 11]. Despite these insights, no meta‐analysis has specifically evaluated RFS and DFS as independent surrogate endpoints for OS in resectable EC. By addressing this gap, the current study provides a focused analysis of these endpoints to clarify their utility, offering critical insights to inform the design of future trials and support timely decision‐making in clinical and regulatory settings.

## 2 Methods

### 2.1 Data Sources and Search Strategy

This review was conducted in adherence with the Preferred Reporting Items for Systematic Reviews and Meta‐Analyses (PRISMA) guidance [12]. Published articles of RCTs were considered if these reported either RFS or DFS as surrogate endpoints and were published between January 1, 2000 and December 31, 2023. These were identified through a search among the PubMed, Web of Science, and the Cochrane Central Register of Controlled Trials databases. PubMed/MEDLINE was searched using the National Library of Medicine controlled vocabulary, Medical Subject Headings (MeSH). Only publications in English were included in the analysis. Search algorithms for each of these three publication data sources are shown in the Supporting Information. Additionally, two studies that fulfilled all eligibility criteria were sourced from the Ajani meta‐analysis [8]. Subsequently, these studies were incorporated into the ensuing analysis.

### 2.2 Eligibility Criteria

Applying the search algorithm, articles were included if these: (1) were designed as an RCT, (2) evaluated the safety or efficacy of therapeutic interventions for EC, (3) used RFS or DFS as primary, co‐primary, or secondary endpoints, and (4) reported hazard ratios (HRs) for both the surrogate endpoint and OS.

Excluded articles included those that: (1) were limited to descriptions of treatment methods or recurrence patterns, (2) focused on nutrition, diet or feeding, habilitation, exercise, or hygiene, (3) were limited to the trial protocol, rationale, design, plan, or management prior to RCT completion, (4) primarily reported the long‐term or short‐term results of the clinical trials, or were either nonrandomized, clinical cases, feasibility studies, safety analyses, benefit or risk assessments, cost‐effectiveness studies, quality assurance, or combined positive scores, (5) primarily investigated non‐EC cancers or related comorbid conditions such as Barrett's esophagus, dysphagia, anemia, and nausea, (6) studied anesthesia applied during esophagectomy and complications after esophagectomy or anastomosis, (7) primarily evaluated quality of life, supportive care or prognostic factors, economic evaluation, endoscopy, tunnel dissection, or screening, (8) used surrogate endpoints other than RFS or DFS, or (9) were review papers or meta‐analyses. Four investigators (U.L.A., O.Q.O., Z.F., and G.R.) reviewed the literature for eligibility.

#### 2.2.1 Publication Bias and Study Quality Assessment

Publication bias was evaluated using funnel plots, which were visually inspected to assess the symmetry of effect sizes plotted against their standard errors. Symmetrical funnel plots indicate a low likelihood of publication bias, whereas asymmetry suggests its potential presence. The funnel plots are included in the Supporting Information.

The quality of the included RCTs was assessed using the Cochrane Risk of Bias Tool (RoB 2) [13, 14]. This tool evaluated bias across five domains: the randomization process, deviations from intended interventions, missing outcome data, measurement of the outcome, and selection of the reported result. Each domain was rated as having low risk, some concerns, or high risk of bias. Two independent reviewers (U.L.A. and Z.F.) conducted the assessments to ensure objectivity and reliability. Any disagreements between reviewers were resolved through discussion, and when consensus could not be reached, a third reviewer (G.R.) was consulted.

#### 2.2.2 Data Extraction and Statistical Analysis

We developed a standardized data extraction form for obtaining relevant information from the retained eligible studies. To evaluate surrogacy using aggregated data, pooled weighted random‐effects HRs for RFS/DFS and OS, after index events (treatment, randomization or registration depending on the study), were determined from the corresponding confidence intervals and subject counts from eligible studies. Heterogeneity was assessed with *I* ^2^ statistics while forest plots were used to visualize the effect size of each study and the pooled estimate. Unweighted linear regression was then used to estimate the correlation between RFS/DFS and OS treatment HRs across studies. To evaluate potential reporting and other biases, funnel plots were created to explore potential asymmetry among the RFS/DFS and OS estimates. Additionally, the linear regression was weighted by sample size as a sensitivity analysis to test the robustness and reliability of the base‐case findings. Since five of the individual studies [15, 16, 17, 18, 19] selected in the current analysis were found to be also selected among those in the Ajani et al. [8] meta‐analysis we applied a second sensitivity analysis by repeating the linear regression estimation between RFS/DFS and OS treatment HRs across studies, but this time after removing these overlapping studies. Stata/MP (ver. 17) was used for all statistical analyses.

## 3 Results

**FIGURE 1:**
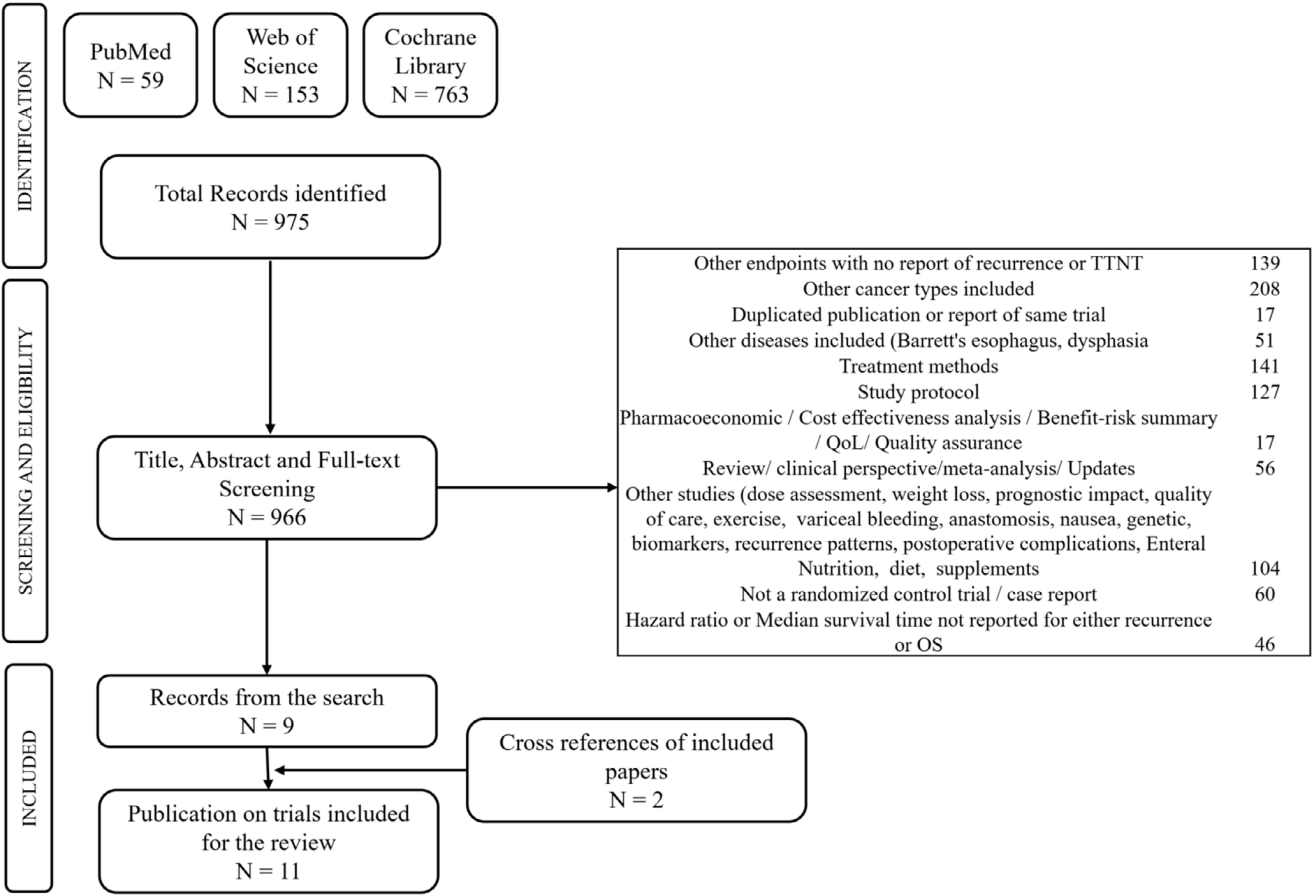
PRISMA flow diagram for trial selection.

A total of 975 published studies were identified after applying the search algorithm; Of these, 966 that satisfied title, abstract, and full‐text screening were evaluated for eligibility. Nine of these studies met all inclusion and exclusion rules. An additional two eligible studies were revealed from cross references, yielding a total of 11 eligible retained studies, comprising 4869 subjects in total. Extracted elements from each study are reported in Table 1. Counts of included and excluded studies and specific reasons for exclusion are shown in Figure 1.

**TABLE 1:** Trials included in this study.

References	Treatment assignment at randomization		Sample size	Hazard ratios; 95% confidence interval; *p*		Endpoints used	Esophageal cancer type	Study site or country
	Treatment	Control		Overall survival	Recurrence/disease‐free survival			
Alderson et al. [19]	Neoadjuvant cisplatin and fluorouracil followed by surgery	Neoadjuvant epirubicin, cisplatin, and capecitabine followed by surgery	897	0·90; 0·77–1·05; *p* = 0·19	0·86; 0·74–1·00; *p* = 0·051	Disease‐free survival (not specifically defined in the paper)	Adenocarcinoma	72 UK hospitals
Chen et al. [20]	Neoadjuvant chemoradiotherapy combined with surgery	Surgery alone	353	1.181; 0.804–1.734; *p* = 0.396	1.541; 1.047–2.268, *p* = 0.028	Recurrence‐free survival (not defined in the paper)	Squamous cell carcinoma	Eight centers in China
Deng et al. [17]	Surgery plus postoperative radiotherapy	Surgery alone	157	0.79; 0.38–1.64; *p* = 0.527	0.53; 0.30–0.94; *p* = 0.030	Disease‐free survival was calculated from the day of R0 surgery to the day of first recurrence or death from any cause or censor	Squamous cell carcinoma	China
Elliott et al. [21]	Neoadjuvant chemoradiotherapy plus surgery	Neoadjuvant chemotherapy plus surgery	2211	1.10; 0.98–1.25; *p* = 0.113	1.18; 1.02–1.37; *p* = 0.023	Distant recurrence‐free survival (not defined in the paper)	Adenocarcinoma	20 high‐volume centers in Europe and North America
Lim et al. [16]	Surgery plus Leucovorin and 5‐fluorouracil (LV5FU2)	Surgery plus LV5FU2 and oxaliplatin (FOLFOX)	62	1.06; 0.44–2.54; *p* = 0.904	1.3; 0.66–2.62; *p* = 0.430	Disease‐free survival was defined as the time from the date of registration to the date of recurrence, death, or last contact.	Squamous cell carcinoma	Multiple centers across South Korea
Mariette et al. [18]	Neoadjuvant chemoradiotherapy followed by surgery	Surgery alone	195	0.98; 0.67–1.44; *p* = 0.92	0.92; 0.66–1.30; *p* = 0.648	Disease‐free survival: Disease recurrence was defined as locoregional (esophageal bed or anastomotic or regional lymph nodes) or metastatic (supraclavicular lymph nodes or distant organs)	Squamous cell carcinoma and Adenocarcinoma	France
Park et al. [22]	Adjuvant durvalumab after neoadjuvant concurrent chemoradiotherapy	Not reported	86	1.08; 0.52–2.24; *p* = 0.85	1.18; 0.62–2.27; *p* = 0.61	Disease‐free survival was calculated as the interval between the date of randomization and the date of either disease recurrence or death due to any cause	Squamous cell carcinoma	South Korea
Sugimura et al. [15]	Neoadjuvant cisplatin and fluorouracil plus docetaxel followed by surgery	Neoadjuvant Doxorubicin followed by surgery	162	0.61; 0.38–0.96; *p* = 0.034	0.55; 0.35–0.86; *p* = 0.012	Recurrence‐free survival was measured from the date of randomization to the date of first evidence of recurrence, death due to any cause, or last follow‐up in patients without recurrence	Squamous cell carcinoma	10 institutions in Japan
Tang et al. [23]	Neoadjuvant chemoradiotherapy followed by minimally invasive esophagectomy	Neoadjuvant Chemotherapy followed by minimally invasive esophagectomy	264	0.82; 0.58–1.18; *p* = 0.28	1.07; 0.71–1.60; *p* = 0.75	Recurrence‐free survival was defined as the time from the date of surgery to the date of first recurrence (local, regional, or distant) or death	Squamous cell carcinoma	10 high‐volume hospitals across China
Yun et al. [24]	Adjuvant chemotherapy (capecitabine and cisplatin)	Surgery only	136	0.85; 0.55–1.34	0.77; 0.49–1.18	Disease‐free survival was defined as the time from surgery to the date of documented disease progression or death. Patients alive without recurrence were censored at the time of the last recurrence surveillance	Squamous cell carcinoma	China at multiple institutions
Zhao e al. [25]	Surgery, two preoperative cycles of paclitaxel, cisplatin, and 5‐fluorouracil (PCF) plus two added postoperative cycles of PCF	Surgery and two preoperative cycles of PCF only	346	0.79; 0.59–0.95; *p* < 0.001	0.62; 0.49–0.73; *p* < 0.001	Relapse‐free survival was calculated from randomization to the first event (i.e., local recurrence, distant recurrence, or death from any cause)	Squamous cell carcinoma	China

Of the 11 retained studies, all incorporated OS endpoints. Of the potential surrogate measures, five studies used RFS endpoints, while six used DFS endpoints that incorporated first of either recurrence or death as the survival event. Squamous cell carcinoma alone accounted for 8 of 11 (73%) of the retained studies. Each study included chemotherapeutic interventions alone or in combination with surgery or radiotherapy. Six studies reported neoadjuvant (55%) and one adjuvant therapy (9%), while the remaining 36% of studies did not report the timing of the chemotherapy used relative to surgery. The various studies enrolled cohorts with largely similar levels of staging, from I to III with no metastatic cancer at entry. Across the studies, 10% of subjects were Stage I upon study entry, 37% were Stage II, and 53% were Stage III.

**FIGURE 2:**
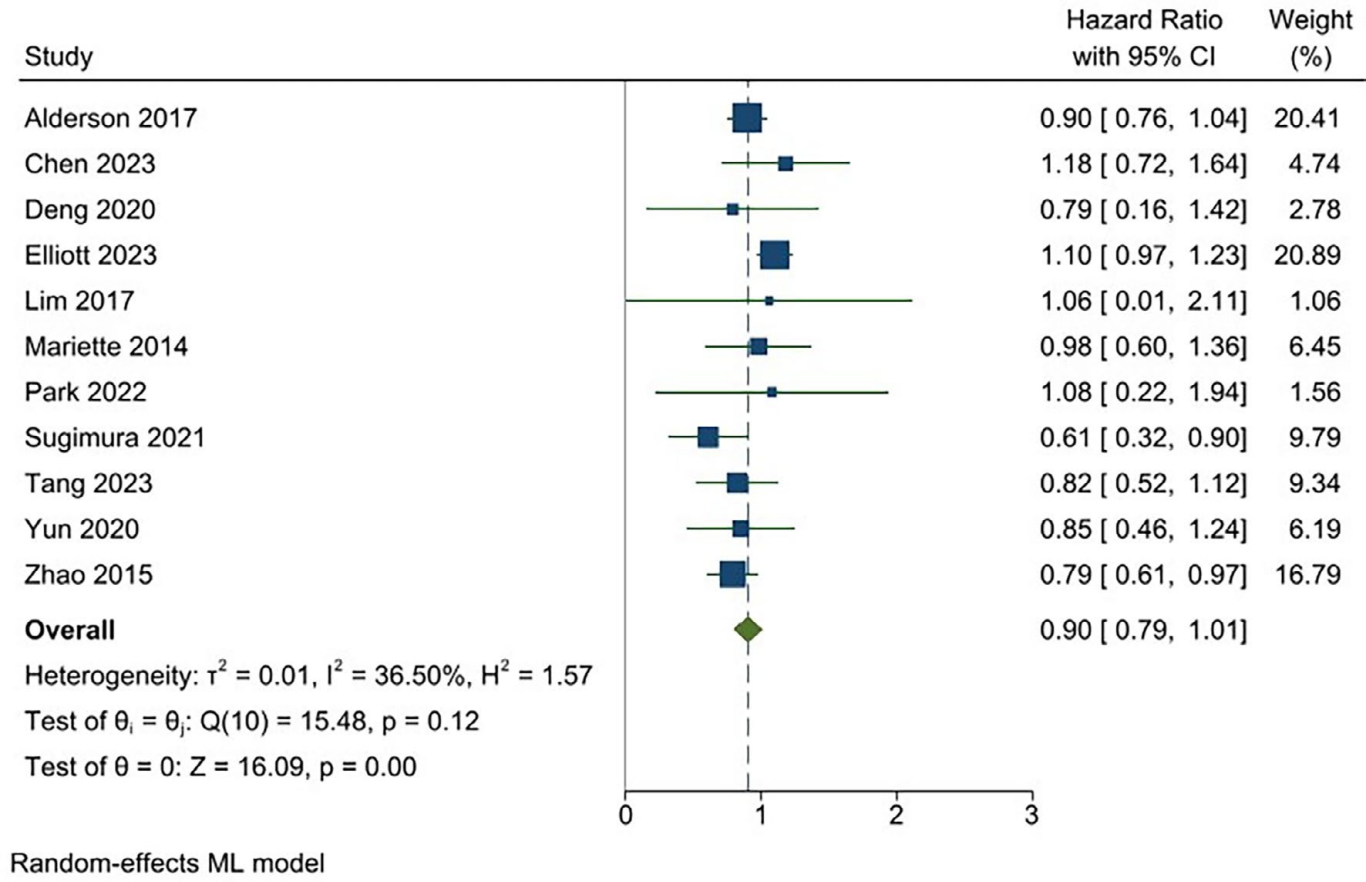
Forest plot showing individual and pooled study‐weighted effects of treatment on overall survival. The pooled HR for overall survival after treatment from the aggregated trials is 0.90 [CI: 0.79–1.01]. CI, confidence interval.

**FIGURE 3:**
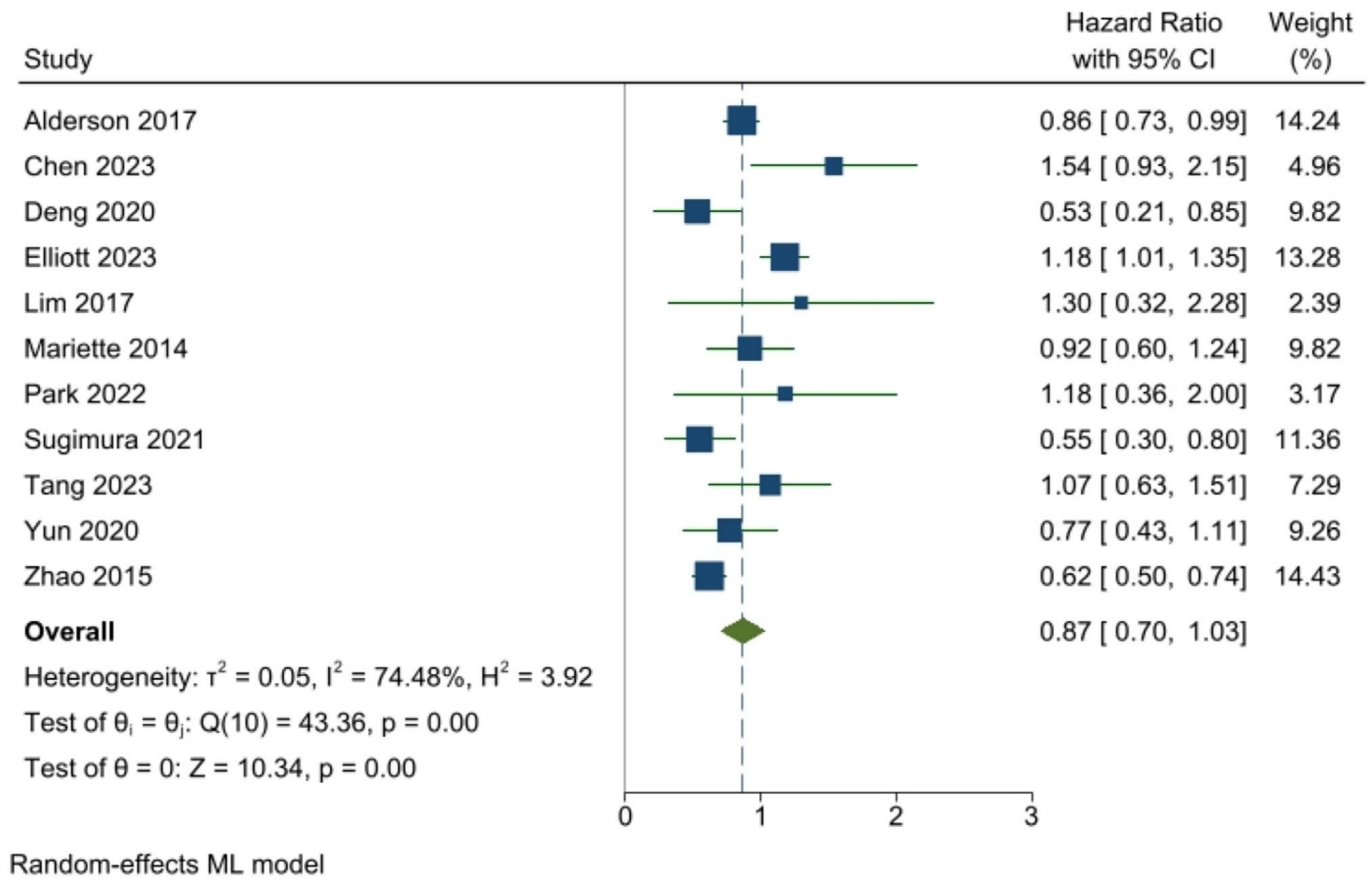
Forest plot showing individual and study‐weighted effects of treatment on recurrence–disease‐free survival. The pooled HR for recurrence–disease‐free survival after treatment from the aggregated trials is 0.87 [CI: 0.70–1.03] CI, confidence interval.

Across the selected studies, treatments generally tended to improve OS efficacy (lower hazard of death) vs. the control, but with substantial variability among the studies. As shown in Figure 2, the pooled analysis of HRs using the weighted random effects model for treatment indicates a higher but non‐significant pooled estimate of OS for the treatment vs. control (HR: 0.90, CI: 0.79–1.01). No significant heterogeneity (*p* = 0.12) in OS was found across the studies. Figure 3 shows a similar pattern of results among studies, but with a slightly higher but non‐significant pooled estimate for RFS/DFS (HR: 0.87, CI: 0.70–1.03). However, significant heterogeneity (*p* < 0.00) for RFS/DFS was found across the studies. Table 2 summarizes the risk of bias for the included studies, highlighting their methodological quality. Most studies demonstrated low risk of bias across key domains, indicating strong methodological rigor. The studies with some concerns (e.g., Mariette et al. [18]; Sugimura et al. [15]) displayed minor methodological limitations, such as issues with blinding or measurement of outcomes. While these do not invalidate the findings, they necessitate a cautious interpretation as they could introduce subtle biases that might influence the results. Importantly, no study was classified as high risk, indicating that all included studies adhered to generally robust methodological standards. The range of HR ratios for DFS/RFS to OS spans from 0.67 to 1.30. Among these ratios, five are below one, while seven are equal to or greater than one.

**TABLE 2:** Summary table for assessing the quality of included studies (Cochrane Risk of Bias (RoB) 2 Framework).

References	Domain 1: randomization process	Domain 2: deviations from intended interventions	Domain 3: missing outcome data	Domain 4: measurement of outcome	Domain 5: selection of reported results	Overall risk of bias
Alderson et al. [19]	Low risk	Low risk	Low risk	Some concerns	Low risk	Some concerns
Chen et al. [20]	Low risk	Some concerns	Low risk	Low risk	Low risk	Some concerns
Deng et al. [17]	Low risk	Low risk	Low risk	Low risk	Low risk	Low risk
Elliott et al. [21]	Low risk	Low risk	Low risk	Low risk	Low risk	Low risk
Lim et al. [16]	Low risk	Low risk	Low risk	Low risk	Low risk	Low risk
Mariette et al. [18]	Low risk	Low risk	Some concerns	Low risk	Low risk	Some concerns
Park et al. [22]	Low risk	Low risk	Some concerns	Low risk	Low risk	Some concerns
Sugimura et al. [15]	Low risk	Some concerns	Low risk	Some concerns	Low risk	Some concerns
Tang et al. [23]	Low risk	Low risk	Low risk	Low risk	Low risk	Low risk
Yun et al. [24]	Low risk	Low risk	Low risk	Some concerns	Low risk	Some concerns
Zhao et al. [25]	Low risk	Some concerns	Low risk	Some concerns	Low risk	Some concerns

*Note:* The table evaluates the risk of bias across five domains as defined in the Cochrane RoB 2 tool, providing an overall judgment for each study. “Low risk” indicates high methodological rigor, while “some concerns” highlights areas needing cautious interpretation.

**FIGURE 4:**
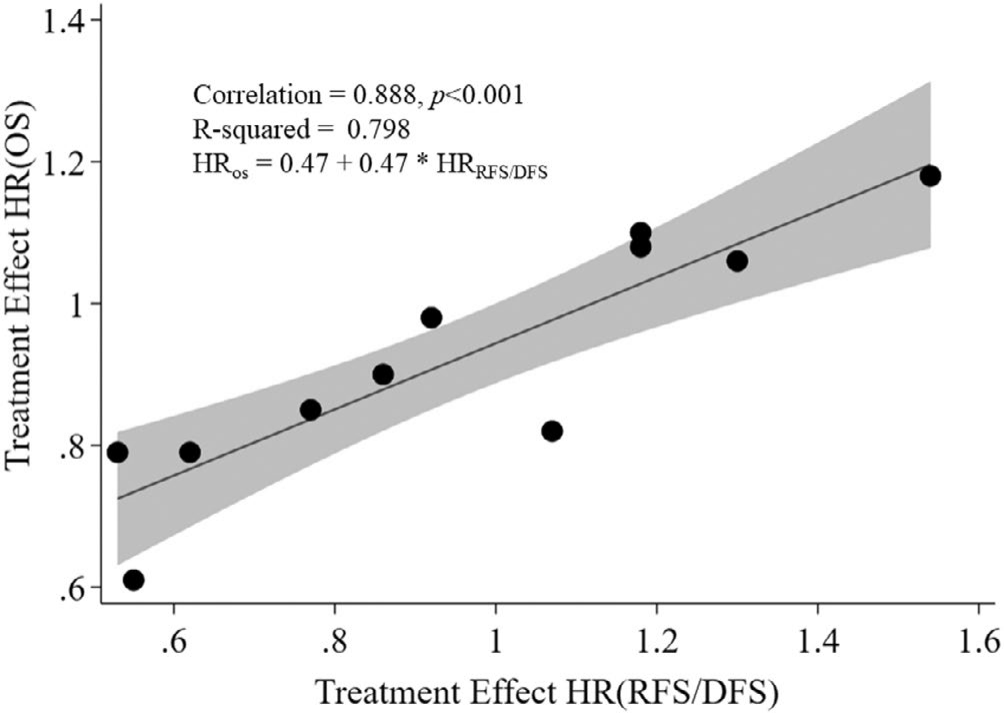
Unweighted regression analysis of treatment effect on overall survival (OS) vs. recurrence‐ or disease‐free survival. Each point represents hazard ratios from each of the studies. The shaded area around the line of best fit represents the 95% confidence interval.

As shown in Figure 4, treatment effects among the 11 individual studies, when unweighted HRs of OS are regressed on RFS/DFS estimates, show a strong and significant correlation (*ρ* = 0.89), with no clear evidence of heteroskedasticity across the range of HR pairs. Although the funnel plot for OS indicates no significant publication bias (all studies fall within the triangular 95th‐percentile region of standard errors), four of the studies in the funnel plot for DFS/RFS fall out of that region, indicating potential publication bias for studies reporting this surrogate endpoint, as shown in the Supporting Information (SI Figures S1 and S2, respectively, for OS and DFS/RFS).

The results of the sensitivity analysis for the linear regression weighted by sample size remained strong and statistically significant (*ρ* = 0.86, *p* < 0.001). After removing the five studies that were also used in the Ajani et al. [8] meta‐analysis the sensitivity analysis focused on the remaining studies. This analysis compared the findings of the weighted regression analysis by sample size with those of the unweighted regression model of treatment effects on OS vs. RFS/DFS survival. The correlation coefficients remained strong and significant for the studies in the current analysis that were not included in the earlier one (*ρ* = 0.86, *p* < 0.001 for the weighted analysis and *ρ* = 0.88, *p* = 0.022 for the unweighted analysis).

## 4 Discussion

In this study we found similarly‐appearing distributions when comparing the forest plots of OS and RFS/DFS. However, the test for heterogeneity, as shown by the *I* ^2^ test, showed a significant level of heterogeneity among the RFS/DFS study HRs that was not observed in the OS plot. This was also confirmed in the funnel plot for RFS/DFS. The latter finding might be due to differences among the selected studies in patient characteristics, clinic sites, or study protocols, but might also be influenced by a potential publication bias, such as when the dissemination of research findings is influenced by the nature and direction of significant positive results [26] or when the design of later studies was modeled on earlier studies that showed positive results [27]. Another possible explanation is that, while death in OS is clearly a much more consistently defined and dated endpoint, the heterogeneity and potential bias seen with the recurrence‐based endpoints of RFS/DFS might have simply been related to the variation, across studies, in how recurrence is defined and measured within each trial.

The ratios of RFS/DFS HRs to OS HRs range from 0.67 to 1.30. Five of these ratios are below 1, indicating a lower hazard for RFS/DFS compared to OS, suggesting a less aggressive predictive capacity. Seven ratios are 1 or higher, indicating an equal or greater hazard for RFS/DFS, suggesting a comparable or more aggressive predictive capacity compared to OS. The strong linear association observed between RFS/DFS and OS from the above meta‐analysis of the 11 selected studies suggests that the generalizability link between these outcomes is robust. This unweighted random‐effects value of 0.89 compares with the significant 0.83 (bivariate random‐effects) and 0.89 (weighted linear regression) correlation values found by Ajani et al. [8] in their meta‐analysis, even though the latter study was dominated by a larger number of eligible PFS vs. DFS studies selected. Our sensitivity analysis findings showed that the strong observed correlation between RFS/DFS and OS remained robust after removing the five studies that overlapped with the studies selected in Ajani et al. [8].

Subject to the limitations described here, RFS/DFS, as shown in this trial‐level meta‐analysis, has the potential for further consideration as a surrogate for OS. Yet, while strong correlation is an expected pre‐requisite for surrogacy, correlation alone is not sufficient for establishing surrogacy. Fleming and DeMets emphasize that a valid surrogate endpoint must not only correlate with the true clinical outcome but also predict the effect of the intervention on the true clinical outcome [28]. This means demonstrating that changes in RFS/DFS directly lead to corresponding changes in OS. Moreover, it is essential to show that the surrogate endpoint consistently predicts OS across different patient populations and treatments and that it captures all relevant effects of the intervention on the true endpoint. Even perfect correlation within randomized groups is insufficient if it does not also meet these stronger conditions for surrogacy [29]. Thus, while promising, further validation of the RFS/DFS endpoint in EC is warranted.

When interpreting our findings, it is important to acknowledge a number of limitations. First, this study relies on a meta‐analysis of published research rather than individual patient‐level analysis; thus, the trial‐level surrogacy supported by a meta‐analysis is not equivalent to individual‐level tests of surrogacy. Our results also rely on a small‐sized literature‐based analysis. It is noteworthy that conducting a similar literature‐based analysis with an ample number of trials in the future poses a challenge due to the limited publication of RCTs in EC each year. The selection of the primary endpoint in a clinical trial should consider the clinical relevance of available endpoints in the study population, as well as the trial purpose and clinical phase.

## 5 Conclusion

This meta‐analysis highlights a strong correlation between RFS/DFS and OS, suggesting that RFS/DFS could serve as a surrogate endpoint in resectable EC. However, while promising, correlation alone does not confirm surrogacy. Validation across diverse populations and treatment settings is essential to establish its predictive value and utility in clinical practice.

## Author Contributions

Conceptualization: Uchechukwu Love Anyaduba. Design and methodology: Uchechukwu Love Anyaduba, Gregory Reardon, Jason Shafrin, and Nazia Rashid. Search, screening of results and data extraction: Uchechukwu Love Anyaduba, Oluwatosin Qawiyy Orababa, Zion Faye, and Gregory Reardon. Data analysis and interpretation: Uchechukwu Love Anyaduba, Oluwatosin Qawiyy Orababa, and Gregory Reardon. Manuscript writing: Uchechukwu Love Anyaduba and Gregory Reardon. Manuscript review and editing: Uchechukwu Love Anyaduba, Gregory Reardon, Jason Shafrin, and Nazia Rashid. All authors have read and approved the final version of this manuscript.

## Ethics Statement

The authors have nothing to report.

## Conflicts of Interest

The authors declare no conflicts of interest.

## Supporting information


**Data S1.** Supporting Information.

## Data Availability

The data that supports the findings of this meta‐analysis was retrieved from published studies and are available in the table of this article.
