# Supplementary material for: Meta‐Analysis of Recurrence‐Free Survival or Disease‐Free Survival as a Potential Surrogate Endpoint for Overall Survival in Esophageal Cancer Trials
Source: Cancer Rep (Hoboken). 2025 May 19;8(5):e70195. doi: 10.1002/cnr2.70195 (PMC12087002; doi:10.1002/cnr2.70195)
Supplement: Supplementary file 1 — Data S1. Supporting Information. [file CNR2-8-e70195-s001.docx]

**Supplemental Material**

Supplemental Material 1. Search algorithm

**Cochrane Central Register of Controlled Trials**

Trials matching esophageal cancer in All Text AND "recurrence" in All Text - with Publication Year from 2000 to 2023, with Cochrane Library publication date Between Jan 2000 and Dec 2023, in Trials (Word variations have been searched)

N = 763

**Web of Science**

((ALL=(Esophageal cancer)) AND ALL=(recurrence) AND ALL=(randomized trial) and Article (Document Types) and 1.95 Gastrointestinal & Esophageal Diseases (Citation Topics Meso) and 1.95.756 Esophageal Cancer (Citation Topics Micro) and English (Languages) and Gastroenterology Hepatology or Surgery or Oncology or Radiology Nuclear Medicine Medical Imaging (Web of Science Categories)

| Timespan: 2000-01-01 to 2023-12-31 (Publication Date)

N = 153

**PubMed**

("Esophageal Neoplasms"[MeSH Terms] AND "neoplasm recurrence, local"[MeSH Terms]) AND ((randomizedcontrolledtrial[Filter]) AND (2000/1/1:2023/12/31[pdat]))

N = 59

### Supplemental Material 2. Methodological Justifications for Risk of Bias Assessment

**Alderson et al., 2017**

- **Randomization:** Allocation was conducted using a computerized minimization program with a random element, stratified by center and tumor stage, ensuring a robust randomization process.
- **Deviations from Intended Interventions:** While blinding of participants and staff was not performed, adherence to the intervention protocol was consistent, minimizing risks from deviations.
- **Missing Outcome Data:** Analysis was performed on an intention-to-treat basis with no significant missing data reported, ensuring validity.
- **Measurement of Outcome:** Pathology assessments were conducted independently with central review for discrepancies; however, the lack of blinding of outcome assessors raises some concerns.
- **Reported Results:** Outcomes were reported transparently, and no evidence of selective reporting was identified.

**Chen et al., 2023**

- **Randomization:** A stratified permuted-block randomization method was used, ensuring proper allocation concealment.
- **Deviations from Intended Interventions:** The blinding of participants and personnel was not described, which could introduce bias, especially for subjective outcomes.
- **Missing Outcome Data:** Follow-up was thorough, with minimal and balanced losses across groups, mitigating the impact of missing data.
- **Measurement of Outcome:** Outcomes were measured systematically, reducing bias; however, the lack of explicit blinding of assessors introduces some concerns.
- **Reported Results:** Reporting was consistent with study objectives, and all prespecified outcomes were addressed.

**Deng et al., 2020**

- **Randomization:** Randomization was conducted using a computer-generated random number system, ensuring appropriate allocation concealment.
- **Deviations from Intended Interventions:** There were no deviations from the planned interventions, and adherence to the protocol was strong.
- **Missing Outcome Data:** Missing data were minimal and handled appropriately without affecting the study’s conclusions.
- **Measurement of Outcome:** Outcomes were clearly defined and assessed objectively by independent clinicians, reducing bias.
- **Reported Results:** All prespecified outcomes were transparently reported, ensuring no evidence of selective reporting.

**Elliott et al., 2023**

- **Randomization:** Randomized allocation was clearly defined and adequately concealed.
- **Deviations from Intended Interventions:** Protocol adherence was strong with consistent interventions reported.
- **Missing Outcome Data:** No significant missing data was reported; losses were minimal and balanced.
- **Measurement of Outcome:** A comprehensive outcome assessment was performed; however, methods ensured low detection bias.
- **Reported Results:** All prespecified outcomes were reported without selective bias.

**Lim et al., 2017**

- **Randomization:** Explicit allocation concealment ensured minimal selection bias.
- **Deviations from Intended Interventions:** Clear adherence to protocol throughout.
- **Missing Outcome Data:** Low attrition rates with balanced losses between groups.
- **Measurement of Outcome:** Objective endpoints reduced bias.
- **Reported Results:** Consistent reporting aligned with study objectives.

### **Mariette et al., 2014**

- **Randomization Process**: Conducted centrally with a minimization technique that ensured equal distribution of patients across stratification factors such as center, histology, stage, and tumor location. This reduces selection bias and supports methodological rigor.
- **Deviations from Intended Interventions**: Adherence to planned treatment protocols was high, and there were no significant deviations, minimizing bias in this domain.
- **Missing Outcome Data**: Some concerns due to attrition, including patients excluded post-randomization for failing eligibility criteria or withdrawing from treatment.
- **Measurement of Outcome**: Standardized procedures were applied for assessing tumor response, but the lack of blinding of assessors raises concerns about potential detection bias.
- **Selection of Reported Results**: All prespecified outcomes were transparently reported, ensuring consistency with the trial objectives.

### **Park et al., 2022**

- **Randomization Process**: The study employed a stratified randomization method using a predefined table to ensure proper allocation concealment. The randomization table was uploaded to an electronic system, reducing selection bias.
- **Deviations from Intended Interventions**: Double-blinding was maintained for both patients and medical staff, ensuring adherence to intervention protocols. The placebo and durvalumab solutions were indistinguishable in appearance.
- **Missing Outcome Data**: Although there were some dropouts due to adverse events, the analysis followed the intention-to-treat principle. However, the study's sample size was smaller than planned, potentially impacting outcomes.
- **Measurement of Outcome**: Objective criteria for tumor assessment were used, with independent evaluation by blinded pathologists for PD-L1 expression. This minimized detection bias.
- **Selection of Reported Results**: All prespecified outcomes were reported, and no evidence of selective reporting was identified.

### **Sugimura et al., 2020**

- **Randomization Process**: Patients were randomized centrally with stratification by institution and clinical N stage, ensuring allocation concealment and minimizing selection bias.
- **Deviations from Intended Interventions**: Adherence to the chemotherapy and surgical protocol was robust across all participating institutions, and blinding was implemented for outcome assessors.
- **Missing Outcome Data**: Minimal data loss was reported, and the intention-to-treat analysis accounted for patients who were lost to follow-up.
- **Measurement of Outcome**: Outcome assessment was blinded, reducing detection bias. However, the potential for subjective interpretation of recurrence patterns introduces slight concerns.
- **Selection of Reported Results**: All prespecified outcomes, including recurrence-free survival (RFS) and overall survival (OS), were reported without evidence of selective reporting.

### **Tang et al., 2023**

- **Randomization Process**: Central randomization was conducted using a computer-generated system with stratification by trial centers, age, and clinical stage. This ensured balanced allocation and robust concealment.
- **Deviations from Intended Interventions**: Interventions were applied as planned, with no significant deviations. Blinding was maintained for outcome assessors, further reducing bias.
- **Missing Outcome Data**: Minimal attrition was observed, with comprehensive follow-up for the intention-to-treat population. However, some patients declined surgery, which could introduce bias if non-random.
- **Measurement of Outcome**: Objective outcomes, including recurrence and survival rates, were assessed systematically. Blinding of assessors reduced detection bias.
- **Selection of Reported Results**: All prespecified outcomes, including 3-year overall survival and recurrence-free survival, were transparently reported.

### **Yun et al., 2020**

- **Randomization Process**: The study utilized centralized randomization across five centers in South Korea with stratification by pathological T stage and N stage. Allocation concealment was adequate.
- **Deviations from Intended Interventions**: Adherence to intervention protocols was high, but blinding was not reported for patients or personnel, raising concerns about subjective outcomes.
- **Missing Outcome Data**: Some patients declined chemotherapy after randomization, but an intention-to-treat analysis was conducted, mitigating concerns.
- **Measurement of Outcome**: Disease-free survival and overall survival were objectively assessed using standardized methods. Lack of blinding for outcome assessors may have introduced detection bias.
- **Selection of Reported Results**: All prespecified outcomes, including recurrence and survival rates, were transparently reported.

### **Zhao et al., 2015**

- **Randomization Process**: The study employed a centralized randomization process with stratification by clinical and demographic factors, ensuring robust allocation concealment.
- **Deviations from Intended Interventions**: The protocol was followed, and interventions were consistent across both arms. However, there was no mention of blinding for participants or personnel, which could introduce bias for subjective outcomes.
- **Missing Outcome Data**: Minimal data loss occurred, and an intention-to-treat analysis was conducted, addressing potential biases from attrition.
- **Measurement of Outcome**: Objective measures, such as relapse-free and overall survival, were assessed. However, the lack of blinding for outcome assessors introduces concerns about detection bias.
- **Selection of Reported Results**: All prespecified outcomes were reported transparently, aligning with study objectives.


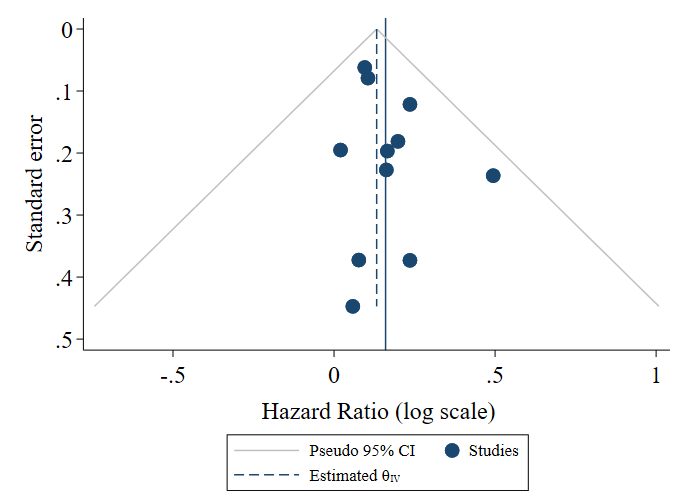
Supplemental Figure 1. Symmetrical funnel plot of individual study overall survival hazard-ratio estimates.

Footnote: Triangular region within dashed line shows 95% confidence range of effect estimates adjusted for sample size of individual studies.


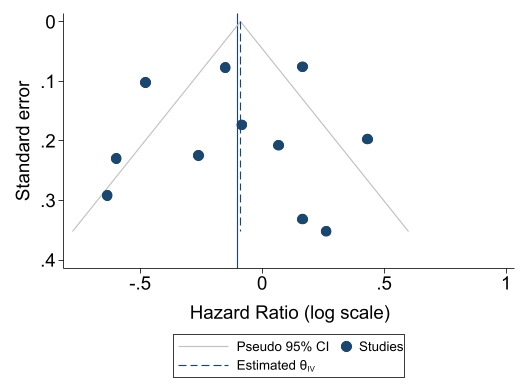


Supplemental Figure 2. Symmetrical funnel plot of individual study recurrence-/disease-free survival hazard-ratio estimates.

Footnote: Triangular region within dashed line shows 95% confidence range of effect estimates adjusted for sample size of individual studies.


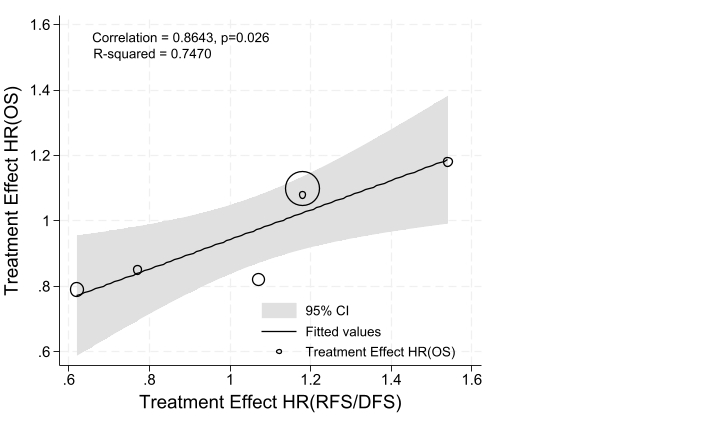


Supplemental Figure 3. Weighted regression analysis of treatment effect on overall survival (OS) versus recurrence- or disease-free survival after removing five studies.

Footnote: In this sensitivity analysis, five of the selected studies from this analysis that were also included in the Ajani et al.(8) meta-analysis have been removed. Each point represents hazard ratios from each of the studies. The shaded area around the line of best fit represents the 95% confidence interval.
